# Exercise and Prediabetes After Renal Transplantation (EXPRED-I): A Prospective Study

**DOI:** 10.1186/s40798-023-00574-8

**Published:** 2023-05-18

**Authors:** Raúl Morales Febles, Domingo Marrero Miranda, Alejandro Jiménez Sosa, Ana González Rinne, Coriolano Cruz Perera, Ana Elena Rodríguez-Rodríguez, Alejandra Álvarez González, Laura Díaz Martín, Natalia Negrín Mena, Cristian Acosta Sørensen, Lourdes Pérez Tamajón, Aurelio Rodríguez Hernández, Federico González Rinne, Aday Dorta González, Eusebio Ledesma Pérez, Alejandra González Delgado, Alberto Domínguez-Rodríguez, Maria del Carmen García Baute, Armando Torres Ramírez, Esteban Porrini

**Affiliations:** 1 Faculty of Medicine, University of La Laguna, La Laguna, Tenerife Spain; 2 Research Unit, University Hospital of Canary Islands, La Laguna, Spain; 3 Nephrology Department, University Hospital of Canary Islands, La Laguna, Spain; 4 Laboratory of Renal Function (LFR), Faculty of Medicine, University of La Laguna, La Laguna, Tenerife Spain; 5 Faculty of Physiotherapy, University of La Laguna, La Laguna, Spain; 6 Central Laboratory, University Hospital of Canary Islands, La Laguna, Spain; 7 Cardiology Department, University Hospital of Canary Islands, La Laguna, Spain; 8 CIBER of Cardiovascular Diseases (CIBERCV), Madrid, Spain; 9 Faculty of Health Science, European University of Canary Islands, La Laguna, Tenerife Spain; 10 Instituto de Tecnologías Biomédicas (ITB), Faculty of Medicine, University of La Laguna, La Laguna, Tenerife Spain

**Keywords:** Renal transplantation, Post-transplant diabetes mellitus, Prediabetes, Exercise and adherence

## Abstract

**Background:**

Post-transplant diabetes mellitus (PTDM) beyond 12 months (late PTDM) is a severe complication after renal transplantation. Late PTDM develops mostly in subjects with prediabetes. Although exercise may have a potential role in preventing late PTDM, there are no previous data on the effect of exercise in patients with prediabetes.

**Material and Methods:**

The design was a 12-month exploratory study to test the capacity of exercise in reverting prediabetes in order to prevent late-PTDM. The outcome was the reversibility of prediabetes, assessed every 3 months with oral glucose tolerance tests (OGTT). The protocol included an incremental plan of aerobic and/or strength training as well as an active plan for promoting adherence (telephone calls, digital technology, and visits). A priori, a sample size cannot be calculated which makes this an exploratory analysis. Based on previous studies, the spontaneous reversibility of prediabetes was 30% and the reversibility induced by exercise will account for another 30%, a total reversibility of 60% (*p* value < 0.05, assuming a potency of 85%). Ad interim analysis was performed during follow-up to test the certainty of this sample calculation. Patients beyond 12 months after renal transplantation with prediabetes were included.

**Results:**

The study was interrupted early due to efficacy after the evaluation of the follow-up of 27 patients. At the end of follow-up, 16 (60%) patients reverted to normal glucose levels at fasting (from 102.13 mg/dL ± 11 to 86.75 ± 6.9, *p* = 0.006) and at 120 min after the OGTTs (154.44 mg/dL ± 30 to 113.0 ± 13.1, *p* = 0.002) and 11 patients had persistent prediabetes (40%). Also, insulin sensitivity improved with the reversibility of prediabetes, compared to those with persistent prediabetes: 0.09 [0.08–0.11] versus 0.04 [0.01–0.07], *p* = 0.001 (Stumvoll index). Most needed at least one increment in the prescription of exercise and compliance. Finally, measures aimed at the improvement of compliance were successful in 22 (80%) patients.

**Conclusion:**

Exercise training was effective to improve glucose metabolism in renal transplant patients with prediabetes. Exercise prescription must be conducted considering both the clinical characteristics of the patients and pre-defined strategy to promote adherence. The trial registration number of the study was NCT04489043.

## Key Points

1. PTDM is a risk factor for major complications: cardiovascular diseases, infections, and renal cell cancer. Strategies to prevent PTDM are limited.
2. Exercise training is an effective alternative to improve glucose metabolism in renal transplant patients. Thus, exercise can be considered, together with diet and medications, as a tool to treat and prevent these complications.
3. A pre-defined strategy to improve compliance must be considered simultaneously with exercise prescription.

## Introduction

Renal transplantation is the best choice for patients with end-stage renal disease [1, 2]. It improves quality of life and reduces mortality compared to dialysis [3, 4]. However, renal transplantation is not free of complications, being cancer, infectious diseases, metabolic, and cardiovascular diseases the most relevant [5–8].

Post-transplant diabetes mellitus (PTDM) may affect about 30% of renal transplant patients [9–20]. The consequences of PTDM are relevant: it is a major risk factor for cardiovascular events, infectious diseases, and renal cell cancer [21, 22]. The incidence of PTDM is bimodal [11]: *early PTDM*, which is observed in an early period after transplantation (the first 3–6 months) and *late PTDM*, that develops in more stable conditions (beyond 12–24 months). Early PTDM affects about 80%, whereas late PTDM 20% of the subjects. Risk factors for PTDM include immunosuppressive therapy and predisposing pre- and post-transplant conditions like age, metabolic syndrome, overweight, obesity, dyslipidemia, prediabetes, and insulin resistance [11, 23].

Several strategies to prevent PTDM have been proposed [13]. Many are related to the selection of patients at risk on the waiting list and the use of a less diabetogenic immunosuppressant [22]. However, this approach pertains to the prevention of early PTDM. On the other hand, limited evidence is available on the prevention of late PTDM. Of note, late PTDM occurs mostly in subjects with post-transplant prediabetes (those with impaired fasting glucose and/or impaired glucose tolerance) [11]. So, renal transplant patients with prediabetes represent the specific population to treat and prevent late PTDM.

In the general population, exercise treatment (aerobic and/or resistance) reduced the incidence of type 2 diabetes mellitus (T2DM) in patients with prediabetes by 40–60% [24–27]. Since PTDM and T2DM share many common characteristics, it is plausible to expect a similar effect in patients with prediabetes after transplantation. However, little evidence is available on the impact of exercise (aerobic and/or resistance) in renal transplantation. In fact, a recent review observed that exercise was an unproven intervention with potential benefits in this population [28]. The few studies available do not help understanding the usefulness of exercise in preventing PTDM, possibly due to the inclusion of patients not at risk (without prediabetes) [29–31].

This was an exploratory design to evaluate the capacity of exercise in reverting prediabetes as a preliminary and necessary step to understand whether exercise can prevent late PTDM in patients at risk.

## Material and Methods

### Design

The protocol (NCT04489043) has been previously published [32]. In brief, this was an interventional prospective study in which renal transplant patients with prediabetes were treated with exercise for 12 months to revert this complication. Only patients with prediabetes beyond 12 months after transplantation with capacity to perform exercise were included. Prediabetes was diagnosed based on fasting glucose levels and OGTTs. Patients were treated with a stepped training intervention, starting with aerobic exercise (brisk walking or cycling) 5 times per week, 30 min/day. Aerobic exercise was gradually increased to 60 min/day or eventually combined with strength exercise in case of persistent prediabetes. The reversibility/persistence of prediabetes was measured with fasting glucose and OGTTs every 3 months. Additionally, other outcomes were evaluated: (a) insulin sensitivity, (b) adherence to exercise, and (c) improvements in metabolic risk factors: obesity, dyslipidemia, and blood pressure.

### Definitions

*Impaired fasting glucose (IFG)* fasting levels of glucose between 100 and 125 mg/dL in more than 2–3 determinations using data from clinical records.

*Impaired glucose tolerance (IGT)* glucose levels at 120 min after an OGTT between 140 and 199 mg/dL [32, 33].

### Patients

Inclusion criteria: (a) renal transplant patients; (b) aged > 18 years; (c) follow-up at least 12 months after transplantation (d) stable renal function; (e) prediabetes: IFG and/or IGT; and (f) ability to perform exercise. The exclusion criteria were: (a) PTDM; (b) diabetes before transplantation; (c) clinical conditions that limit the performance of exercise (clinical instability: active infection, cancer, acute cardiovascular disease, advanced renal disease, pulmonary hypertension, and limb amputations); (d) morbid obesity, body mass index (BMI) ≥ 40; (e) inability to understand the protocol; and (f) severe psychological disease. Finally, subjects were not included based on a positive motivational status or willingness toward exercise.

#### Screening

The selection of prediabetes was as follows: (a) patients with IFG were detected by the evaluation of clinical records, (b) the diagnosis of IGT was an OGTT, since it frequently coincides with normal fasting glucose levels. To perform an OGTT in all non-diabetic patients is not cost-effective. Based on a previous report [33], the presence of metabolic syndrome traits (BMI > 27 kg/m^2^ and high triglyceride levels > 150 mg/dL) was used as a proxy to identify those patients at a higher risk for having IGT.

### Procedures

#### OGTT

After a 10–12 h overnight fast, the patient drank a solution of 75 g/200 mL glucose. Serum samples were taken before (0 min) and after (120 min) the patient took the solution to measure glucose. The test was performed at baseline (screening) and every 3 months to the end of the study (month 12) to evaluate the reversibility, relapse, or persistence of prediabetes. The OGTT was not performed in patients who developed PTDM.

To perform the OGTT, patients had to be stable without conditions that could induce transient hyperglycemia, insulin resistance (infections, acute rejection, cardiovascular disease, and acute kidney injury) [11]. The presence of any of these conditions postponed the test for 1–3 months after full recovery. Also, serum samples were collected to measure insulin before and after the OGTT to calculate insulin sensitivity indexes: Matsuda and Stumvoll [34–36].

#### Pre-treatment Evaluation

All patients underwent a personal interview to evaluate global physical activity or sedentary behavior with the General Physical Activity Questionnaire (GPAQ) [37]. Also, the importance of exercise, its relationship with PTDM, and barriers to exercise (psychological, logistics, and cognitive) were discussed.

#### Anthropometric Measures

At baseline and every 3 months weight, waist circumference, hip circumference, BMI, and waist to hip ratio were measured [38–40].

#### Analytics

At pre-specified time points (baseline, 3, 6, 9, and 12 months), blood samples were taken to determine: hemogram, creatinine, glycated hemoglobin (HbA1c), total, low-density lipoprotein (LDL), and high-density lipoprotein (HDL) cholesterol, triglycerides, uric acid, and other parameters.

### Exercise Treatment

The protocol includes a stepped incremental plan of exercise both aerobic and strength. Changes in frequency, intensity, and duration of aerobic exercise, as well as the addition of strength training depended on the recovery, persistence, or recurrence of prediabetes.

#### Aerobic Exercise

At baseline all patients started with aerobic exercise: brisk walking or cycling. The intensity was moderate (50–70% heart rate), 30 min/day, and 5 times per week. No more than two consecutive days without exercise were allowed [41–45]. Changes in brisk walking or cycling prescription were based on the persistence or recurrence of prediabetes during follow-up.

- *At 3 months* in the case of persistent prediabetes, aerobic exercise was increased to 45 min/day and 5 times per week. When prediabetes reverted, prescription remained the same as in baseline.
- *At 6 months* if prediabetes persisted, exercise increased to 60 min/day and 5 times per week. If prediabetes reverted, the prescription of exercise remained stable: 45 min/day and 5 times per week. In patients without prediabetes since month 3, baseline prescription was not changed.
- *At 9 months* in patients without prediabetes since month 3 or 6, previous prescription was unchanged: 30 or 45 min/day and 5 times per week, respectively. In the cases with relapsing prediabetes, exercise was increased according to previous prescription, from 30 to 45 min/day 5 times per week or from 45 to 60 min/day 5 times per week. However, in patients with persistent prediabetes from month 3 to 9, aerobic exercise was practiced 45 min/day and 3 times per week, adding strength exercise. Detailed information can be found in the published protocol [32].

#### Strength Exercise

Patients were trained and evaluated in the renal unit of the hospital by the physiotherapist to tailor and select each type of exercise according to individual capacities. The intensity was moderate (an exercise that can be repeated no more than 15 times) to vigorous (an exercise that can be repeated no more than 6–8 times). Patients practiced at least 8–10 exercises of 1–3 sets near failure on every exercise [44] 2 times per week on non-consecutive days with a rest period of at least 1 min between sets when the intensity was moderate and at least 3 min when the intensity was vigorous. Functional exercises were selected and reviewed by the physiotherapist depending on the individual capacities [44, 45] including chest press, lateral pull downs, shoulder press, arm curls, triceps extension, sit-ups, deadlift, leg press, and squats. This prescription was set on top of the aerobic exercise training from 9 months on.

### Evaluation of Adherence

To evaluate and promote compliance, a specific protocol was designed, including a fixed and a flexible plan. The fixed plan included the use of a bracelet activity tracker, telephone calls, and a regular plan of visits to the center. The flexible plan was pre-specified for patients with inadequate adherence and included both extra telephone calls and visits to the center [46, 47]. For the scope of this study, adherence was classified a priori in 3 groups depending on the accomplishment to training: (a) *acceptable: *≥ 70%; (b) *moderate:* 40–70%, and (c) *bad*: ≤ 40% [48] covering three major aspects:

*(A) Digital monitoring* every patient received an activity tracker (Mi Global Home). Aerobic exercise was recorded in the device, which could be accessed and reviewed by the physiotherapist to evaluate the plan and to have data on the amount and quality of exercise. Also, with this device the patient could receive feedback and support from the physiotherapist. Finally, each session was recorded in a database.

*(B) Phone calls* a scheduled plan of phone calls was designed to provide support to patients on different aspects: the performance of exercise, possible injuries, barriers to exercise, adaptation to training, improvements, limitations (physical and mental), doubts among others. The calls also provided an opportunity to identify a variety of obstacles to lifestyle changes and to discuss behavioral approaches to improve specific problems. In the fixed plan, patients were contacted once per week during the first 3 months, twice per month from 3 to 6 months, 1 every 3 weeks from 6 to 9 months, and 1 per month in the last 3 months. In the flexible plan, patients with bad–moderate adherence were contacted once per week, independently of the period. All telephone calls, those scheduled or extra, were recorded in a database.

*(C) Visits* participants had to attend a face-to-face meeting every 3 months in the research unit of the hospital. Extra-visits were scheduled in patients with moderate–bad adherence. Visits were focused to evaluate aspects related to exercise or prediabetes and PTDM such as: achievements; weight and glucose levels reduction; behavioral/motivational aspects; possible injuries and adaptation to exercise. Finally, patients received information about healthy lifestyle advice including diet. Sessions lasted from 25 to 40 min each. Visits, regular or extra, were also recorded in a database.

### Statistical Analysis and Sample Calculation

There is almost no evidence available on the effect of exercise on renal transplanted patients with prediabetes or PTDM. Moreover, a possible effect of exercise in preventing the evolution from prediabetes to PTDM has not been specifically investigated. This limits the possibility of calculating a sample size of a study.

Accordingly, this study was designed to test the capacity of physical exercise to revert prediabetes as necessary step to design a clinical trial to prevent PTDM (EXPRED-II). In a previous study of our group, [11] it was observed that prediabetes may spontaneously revert to normal glucose metabolism in 25–30% of the cases. Clearly, studies aimed at the prevention of PTDM in patients with prediabetes, must consider this spontaneous reversibility in the calculation of the sample size. In consequence, to be considered as effective, any tested intervention designed to prevent PTDM in subjects with prediabetes must have an effect significantly larger than the spontaneous reversibility rate of prediabetes. To be sure that reversibility of prediabetes could be attributes to exercise, it was established a priori that the treatment must double the spontaneous reversibility of prediabetes: from 30 to 60%. Assuming that in 60 cases with prediabetes, the spontaneous reversibility could be of 30% (*n* = 18) and the reversibility induced by exercise will account for another 30% (*n* = 18); the total reversibility will be 60%. This difference, 60% versus 30%, would be significant with a *p* value < 0.05, assuming a potency of 85%. Considering an expected drop out of 20%, the total number of cases necessary to include will be 72 patients. Ad interim analysis was performed during follow-up to test the certainty of this power calculation. Statistical analysis was carried out with SPSS (IBM Corp. Released 2017. IBM SPSS Statistics for Windows, Version 25.0. Armonk, NY), and sample size was calculated with GRAMM0 V. 7.12 (IMIM, Spain).

### Sensitivity Analysis

Firstly, to study the effect of gender in response to exercise training. Also, to analyze the incidence of partial reversibility, defined as the persistence of prediabetes but with major reductions in glucose levels either fasting (about 20 mg/dL) or after an OGTT (about 40 mg/dL), that is from 125 to 101 or from 190 to 151 mg/dL.

### Adverse Events

All adverse events associated with exercise training were recorded.

## Results

An ad-interim analysis with 27 patients who reached the end of follow-up showed that 16 subjects (60%) with prediabetes reverted to normal glucose metabolism. This rate was two times higher than the reversibility rate found in a prospective study of our group where 18 of 60 (30%) patients with prediabetes had spontaneous reversibility [11]. This difference, 60% versus 30%, was significant with a *p* value < 0.05, assuming a potency of 85%. Accordingly, the study was interrupted early due to efficacy.

### Patients: Baseline Characteristics

**Fig. 1:**
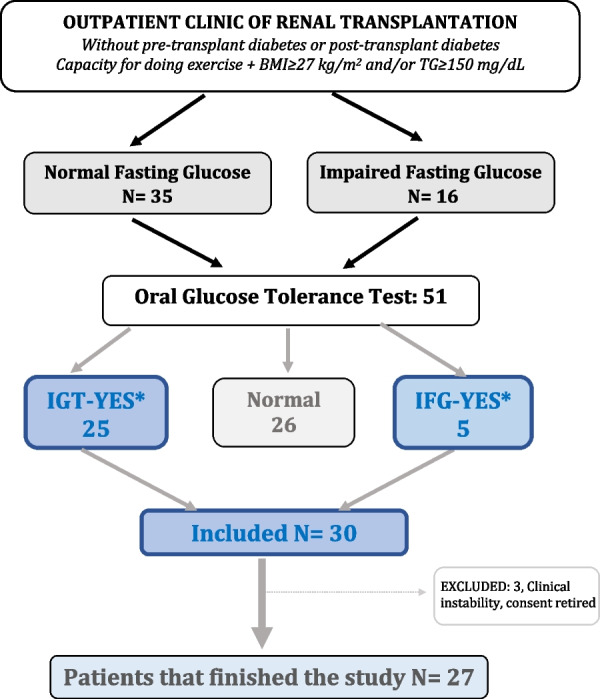
Flowchart of the study showing patients screened, included, excluded and those who reached the end of follow-up. *IGT* impaired glucose tolerance, *IFG* impaired fasting glucose. *Patients with concomitant IGT and IFG: 12

A total of 51 patients were screened, 30 were included and 21 excluded of the study (Fig. 1). All patients were of Caucasian origin. The only cause of exclusion after screening was the lack of prediabetes based on an OGTT. Of the 30 patients included, 3 were excluded during follow-up due to clinical conditions not related to exercise and 27 completed the study (Fig. 1). Mean age was 54.2 ± 9.6 years and 67% were men. Half of the patients were obese and most had overweight. IGT or IFG alone was found, respectively, in 13 patients (48%) and 5 (19%) patients, and the combination of both in 9 (33%). Baseline renal function was comparable between groups (Table 1). All the 27 patients were on prednisone and tacrolimus during the study; 20 subjects were on mycophenolate and 7 on everolimus.

**Table 1:** Baseline and 12 months: characteristics of the patients who finished the 12-month follow-up

	Overall	Prediabetes 12 months	Normal 12 months
*N*	27	11	16
*Baseline*			
Age (years)	54 ± 10	54 ± 10.4	54.3 ± 9.4
Gender (male-%)	18 (67)	8 (73)	10 (63)
Weight (kg)	88.4 ± 19.7	92.4 ± 15	85.6 ± 22.4
Height (cm)	168.3 ± 11.4	168.7 ± 11	168 ± 12
BMI (kg/m^2^)	30.9 ± 4.7	32.5 ± 4.2	30 ± 5
> 30 (kg/m^2^)	17 (63)	8 (73)	9 (56)
Hip (cm)	104.1 ± 14.2	106 ± 12.4	102.8 ± 15.6
Waist (cm)	107.2 ± 12	111 ± 12	104.3 ± 11.3
Waist to hip ratio	1.04 ± 0.11	1.01 ± 0.1	1 ± 0.1
*OGTT*			
Glucose 0’ (mg/dL)	104.1 ± 12.1	107.1 ± 13.4	102.13 ± 11
Glucose 120’ (mg/dL)	168.1 ± 51.5	188.1 ± 69.4	154.44 ± 30
IFG *n*, %	16 (59)	7 (64)	9 (56)
IGT *n*, %	22 (81)	10 (91)	12 (75)
IFG and IGT *n*, %	11 (41)	6 (55)	5 (31)
*Insulin sensitivity*			
Matsuda index	4.2 [2.9–6.4]	4.2 [2.5–7.8]	4.3 [2.8–6.7]
Stumvoll index	0.06 [0.04–0.08]	0.05 [0.02–0.08]	0.06 [0.04–0.08]
Systolic BP (mmHg)	140.9 ± 17.1	141 ± 14.8	140.9 ± 18.4
Diastolic BP (mmHg)	78.4 ± 8.3	82.8 ± 4.6	75.3 ± 8.4^a^
Total Chol (mg/dL)	177.6 ± 28.8	169.8 ± 32.6	182.9 ± 25.7
Triglycerides (mg/dL)	151.4 ± 61.9	147.7 ± 65.9	153.9 ± 61.1
Previous CV events	7 (25%)	4 (36%)	3 (18%)
Family history of DM	6 (22%)	2 (9%)	4 (25%)
HbA1c (%)	5.9 ± 0.3	6.1 ± 0.4	5.8 ± 0.2
Uric acid (mg/dL)	5.8 ± 1.6	6 ± 1.5	5.6 ± 1.7
Creatinine (mg/dL)	1.30 ± 0.3	1.32 ± 0.3	1.29 ± 0.3
Estimated GFR (mg/dL)	58 ± 14.3	57.6 ± 14.8	58.4 ± 14.3
*12 months*			
Weight (kg)	83.3 ± 19.4	87.4 ± 14.3	83.3 ± 22.2
BMI (kg/m^2^)	29.1 ± 4.5	30.7 ± 3.6	28.0 ± 4.8
> 30 (kg/m^2^)	14 (52)	8 (73)	6 (37)
Hip (cm)	95.6 ± 14.3	99.0 ± 12.5	93.2 ± 15.4
Waist (cm)	98.7 ± 9.9	101.5 ± 7.7	96.7 ± 10.9
Waist to hip ratio	1.04 ± 0.1	1.04 ± 0.1	1.05 ± 0.1
*OGTT*			
Glucose 0’ (mg/dL)	90.4 ± 9.8	95.82 ± 9.8	86.75 ± 6.9^b^
Glucose 120’ (mg/dL)	127.5 ± 31.2	153.3 ± 27.4	113.0 ± 23.1^c^
*Insulin sensitivity*			
Matsuda index	6.1 [4–10.1]	2.8 [2.4–5.3]	6.5 [6–12]^d^
Stumvoll index	0.08 [0.05–0.09]	0.04 [0.01–0.07]	0.09 [0.08–0.11]^e^
SBP (mmHg), range	127.8 ± 12.1	129.9 ± 7.7	126.3 ± 14.5
DBP (mmHg), range	73.4 ± 7.3	77.6 ± 6.7	70.5 ± 6.4
Total Chol (mg/dL)	166 ± 28.4	157 ± 28.2	172.5 ± 27.6
Triglycerides (mg/dL)	113 ± 45.3	138 ± 55.9	95.7 ± 26.6^f^
HbA1c (%)	6 ± 0.4	6.2 ± 0.5	5.8 ± 0.2^ g^
Uric acid (mg/dL)	5.9 ± 1.7	6.3 ± 1.5	5.5 ± 1.7
Creatinine (mg/dL)	1.27 ± 0.3	1.35 ± 0.3	1.21 ± 0.3
Estimated GFR (mg/dL)	60 ± 14.9	56.2 ± 13.8	62.6 ± 15.4
*Medication*			
Prednisone (*n*-%)	27 (100%)	11 (100%)	16 (100%)
Tacrolimus (*n*-%)	27 (100%)	11 (100%)	16 (100%)
Mycophenolate (*n*-%)	20 (75%)	8 (72%)	12 (75%)
Everolimus (*n*-%)	7 (25%)	3 (27%)	4 (25%)
Post-tx years	5.6 [2.2–12.5]	5.6 [2.2–11.6]	6 [2–18.7]

Overall and those who reverted to normal glucose metabolism or persisted with prediabetes. *p* values: a = 0.017; b = 0.006; c = 0.002; d = 0.001; e = 0.002 f = 0.016; g = 0.07

*BMI* body mass index, *IFG* impaired fasting glucose, *IGT* impaired glucose tolerance, *BP* blood pressure, *CV* cardiovascular, *DM* diabetes mellitus, *HBA1c* glycated hemoglobin, *GFR* glomerular filtration rate

### Outcome

**Fig. 2:**
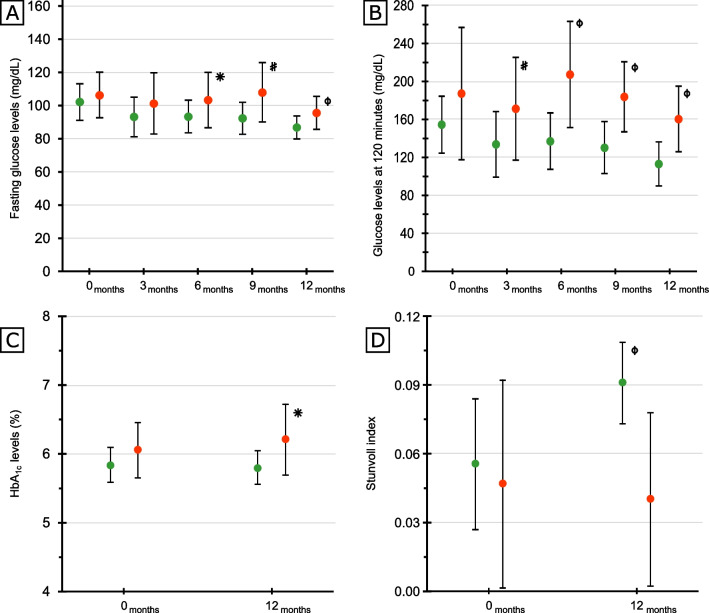
Glucose, hemoglobin, and insulin sensitivity (Stumvoll index) changes overtime in patients with persistent prediabetes or normoglycemia at the end of the study. *p* values: *ϕ* =  ≤ 0.01; ^#^˂0.05 and *0.07

At the end of follow-up, 16 (60%) patients reverted to normal glucose levels both at fasting and at 120 min of the OGTT, whereas 11 (40%) had abnormal levels of glucose: 9 prediabetes and 2 developed PTDM (7%). Ten of 16 (63%) reverted at 3 months, 5 (32%) at 6 months, and 1 (5%) at 9 months (Fig. 2).

#### Metabolic and Anthropometric Parameters

At 12 months, triglyceride levels were lower in patients with reverted prediabetes compared with those with persistent hyperglycemia at 12 months (Table 1). At baseline, insulin sensitivity was comparable between groups (Table 1). At the end of follow-up, insulin sensitivity improved in patients in whom prediabetes reverted and remained stable in those with persistent prediabetes: 0.09 [0.08–0.11] versus 0.04 [0.01–0.07], *p* = 0.001 (Stumvoll index, Table 1 and Fig. 2); 6.5 [6.0–12] versus 2.8 [2.4–5.3], *p* = 0.002 (Matsuda index, Table 1). No significant changes were found in weight, cholesterol, blood pressure, waist to hip ratio at 12 months. .

### Changes in Exercise Prescription

**Fig. 3:**
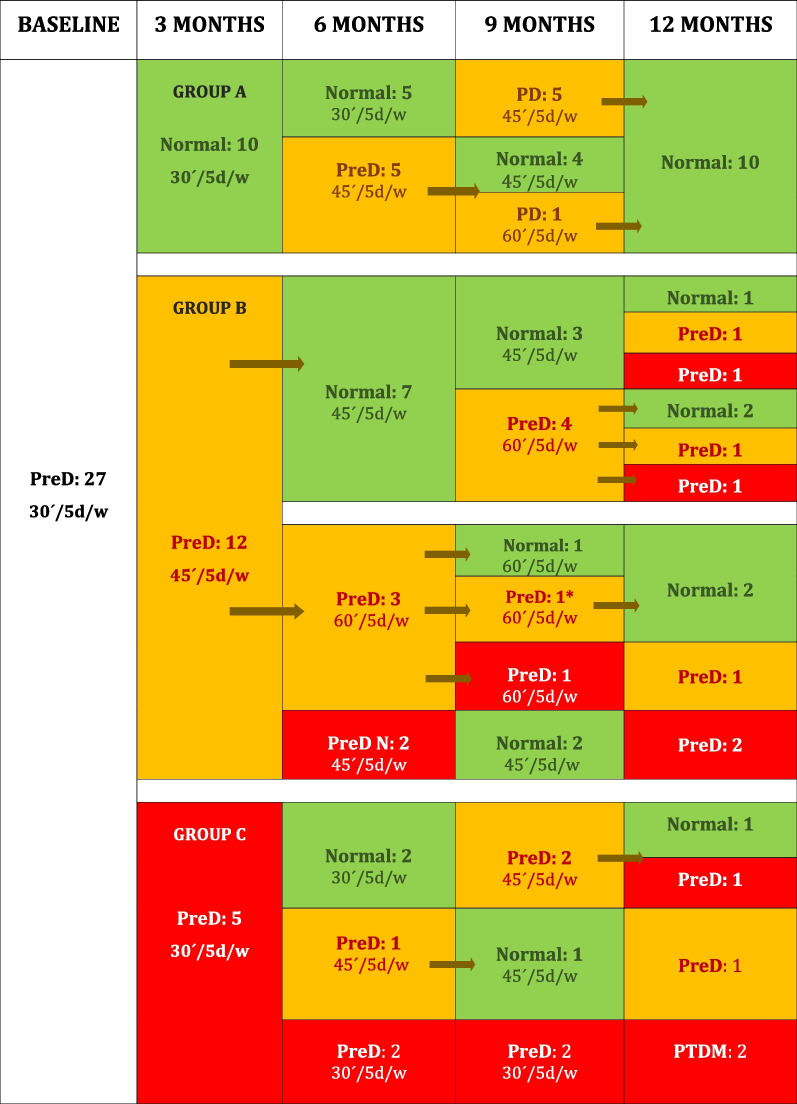
Evolution of prediabetes and exercise prescription from month 3 to the end of follow-up. *Strength training; w: week; d: days; pred: prediabetes; PTDM: post-transplant diabetes mellitus. Green: normal glucose metabolism with good adherence; orange: prediabetes with moderate adherence and red: prediabetes or PTDM with bad adherence. The arrows indicate the changes in exercise prescription and the effect of the increment

To facilitate the description of exercise changes through the study, patients were classified at month 3 in those with (A) normal glucose levels with good–moderate adherence, (B) persistent prediabetes with good–moderate adherence, and (C) persistent prediabetes with bad adherence to exercise (Fig. 3).

#### Group A: Normal Glucose Levels and Good–Moderate Adherence at Month 3 (*n* = 10)

Baseline prescription (30 min, 5 days/week) was not modified (Fig. 3). At month 6, half of the patients had normal glucose levels, whereas prediabetes relapsed in the other half. Thus, exercise was increased to 45 min, 5 days/week in those with relapsing prediabetes (Fig. 3—changes indicated with arrows). At month 9, in the 5 patients without prediabetes at 6 months, prediabetes relapsed, and so, exercise was increased to 45 min, 5 days/week. In the group of 5 patients in whom prediabetes relapsed at month 6, the disease reverted in 4 patients and persisted in 1. So, only in this patient, the prescription was increased to 60 min, 5 days/week. Finally, at 12 months, all subjects in this subgroup showed normal glucose levels. All had good or moderate adherence to exercise during the study (Fig. 3).

#### Group B: Persistent Prediabetes and Good–Moderate Adherence at Month 3 (*n* = 12)

Exercise was increased to 45 min, 5 days/week in all patients (Fig. 3—changes indicated with arrows). At month 6, prediabetes reverted in 7 patients and persisted in 5. Later, at 9 months, in the sub-group of 7 patients who had shown good to moderate adherence, 3 had normal glucose levels and in 4 prediabetes relapsed. In these 4 cases, exercise was increased to 60 min, 5 days/week (Fig. 3—changes indicated with arrows). At month 12, 3 subjects had normal glucose levels and 4 had prediabetes, 2 of them with bad adherence to exercise. *In the group of 5 subjects with persistent prediabetes at month 6*, 3 had good–moderate and 2 bad adherence to exercise. In the former group (*n* = 3), exercise was increased to 60 min, 5 days/week, whereas in the latter (*n* = 2), only adherence-promoting strategies were performed. At month 9, prediabetes reverted in 3 cases, all showing good–moderate adherence and persisted in 2: 1 patient with bad and the other with good adherence. In the first patient, adherence-promoting strategies were implemented and in the other, strength training was prescribed. Finally, at 12 months in this subgroup of 5 subjects, prediabetes reverted in 2, relapsed in 2, and persisted in 1 with bad adherence.

#### Group C: Persistent Prediabetes with Bad Adherence to Exercise at Month 3 (*n* = 5)

Incremental adherence-promoting strategies were applied in all subjects (Fig. 3). These measures were ineffective in 2 cases during the study. Of the remaining 3 cases, all have good–moderate adherence at 6 months: 2 had normal glucose levels and 1 prediabetes. In the latter, exercise prescription was increased. At month 9, this patient reverted, whereas the 2 patients that had reverted at month 6 relapsed, and so, the prescription was increased to 45 min, 5 days/week. Finally, at 12 months, in this group only 1 patient had normal glucose metabolism, while the other 4 patients had abnormal glucose metabolism: 2 had prediabetes and 2 developed PTDM (Fig. 3).

### Compliance to Exercise Treatment

**Fig. 4:**
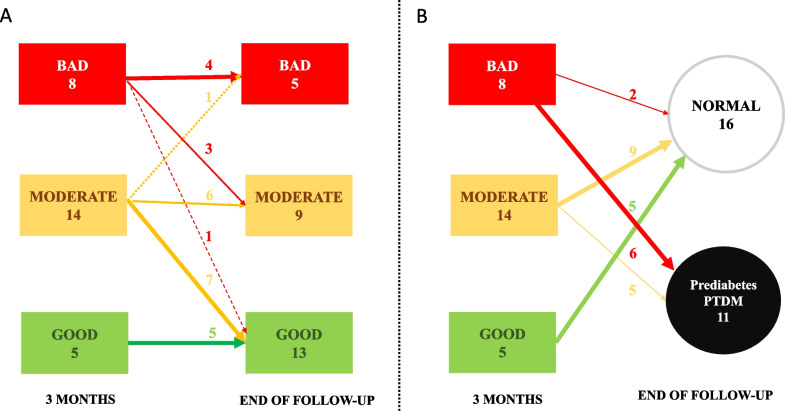
Adherence to exercise: **A** changes in adherence from 3 to 12 month; **B** association between adherence and reversibility/persistence of prediabetes. PTDM: post-transplant diabetes mellitus

In general, active measures were implemented in 22 patients out of 27. Patients were classified according to the level of adherence (Fig. 4).

#### Bad Adherence

Eight patients had bad adherence to exercise. Extra telephone calls and visits were implemented. In 4 patients, this approach was effective, and adherence improved (Fig. 4). In the remaining 4 cases, extra increments in the adherence plan led to better adherence only in 2. Finally, 2 patients did not improve despite several increments in the plan. A total number of 49 extra telephone calls (mean per patient: 8 IQR: 4–11) and 29 visits (mean per patient: 3 IQR: 3–5) were set-up in this group. Thus, extra measures to improve compliance were effective in 4 of 8 (50%) patients during follow-up (Fig. 4). However, only 2 (25%) cases finished the study with normal glucose levels (Fig. 4).

#### Moderate Adherence

Fourteen patients had moderate adherence to exercise. Extra telephone calls and visits (flexible plan) were implemented. These measures improved compliance in 7 (50%) (Fig. 4). In the other 7, this intervention was reinforced two or more times and these changes were effective in 6 (80%) (Fig. 4). A total number of 44 extra telephone calls (mean per patient: 2 IQR: 1–4) and 22 visits were set-up in this group (mean per patient: 1 IQR: 1–2). Finally, at the end of the study, 9 out of 14 (65%) cases with previous moderate adherence reverted to normal glucose metabolism (Fig. 4).

### Sensitivity Analysis

Partial reversibility was observed in 4 (15%) patients, so partial and total reversibility together reached 75%. There were differences between men and women on the response to treatment. In men, 11 of 18 (61%) prediabetes reverted, whereas 5 out of the 9 (55%) women had no prediabetes at 12 months. Considering total and partial response together, 15 of 18 (83%) men and only 5 of 9 (55%) women showed an improvement in glucose levels.

### Adverse Events

Only two exercise-related complications were detected: one iliotibial band syndrome and one plantar fasciitis. Both were treated and solved. These pathologies did not jeopardize the continuity of these patients in the study.

## Discussion

A plan of exercise training was able to reverse prediabetes in stable patients with at least 12 months after renal transplantation. To achieve this goal, it was planned a flexible approach adjusted to individual physical capacities and the evolution of glucose levels with an active strategy for promoting adherence. Of relevance, the latter proved to be a crucial aspect of exercise treatment.

This study was designed to evaluate the impact of exercise in prediabetes in renal transplanted patients at risk for late PTDM. It was exploratory in nature since there is no sufficient evidence on the role of exercise in late PTDM prevention, particularly in subjects at risk for this disease. Thus, our results must be confirmed in further investigations. Our main aim was to evaluate the reversibility of prediabetes by exercise as a first step in the understanding of the effect of this specific treatment in preventing late-PTDM.

Since this complication develops beyond 12–24 months after transplantation and occurs mostly in patients with prediabetes, only subjects with this condition were included. For exercise prescription, current guidelines and studies made in the general population with prediabetes were followed [24–27, 42–45]. Our main finding was thate prediabetes was reverted by a plan of physical exercise. Previous studies showed that prediabetes after transplantation may spontaneously revert by 20–30% on follow-up [11]. In the present intervention study, the reversibility rate of prediabetes by exercise was two times higher than the spontaneous reversibility rate: 60%. Moreover, combining data from total and partial reversibility rates together indicated that 75% of the patients reverted. This finding is in line with studies from the general population showing that exercise training can prevent the evolution from prediabetes toward diabetes [24–27]. Although our outcome was not the incidence of diabetes but the reversibility of prediabetes, it may still be considered that exercise is a useful tool to reduce hyperglycemia after transplantation in patients at risk. In any case, the effect of exercise in preventing late PTDM must be tested in ad hoc designed studies.

Another finding of the study was that the prescription of exercise had to be increased in dose, intensity, frequency, and duration during follow-up in all patients. This clearly means that baseline prescription of exercise was not enough to revert prediabetes. In patients without previous history of exercise training, caution is needed in the implementation of exercise to avoid adverse events and low adherence. Clearly, the implementation of exercise must consider a balance between intensity, tolerability, sustainability, and efficacy. In line with these ideas, the protocol started with moderate intensity (30 min/day of brisk walking 5 days a week). Although for many patients this plan was tolerable, for others, particularly those with lower physical capacities it could have been considered excessive. However, it is worth mentioning that very few subjects suffered from injuries related to the prescription (only two cases). The plan was defined as incremental, a decision made a priori, taking into account the efficacy of the prescription (the reversibility of hyperglycemia). In the same line, the plan was incremental to cope with the known accommodation effect of exercise. Thus, a “tailored-made exercise prescription” is preferable than a “one-size-fits-all” approach. Finally, the intervention proposed was simple, easy, tolerable, and with reduced costs, which may facilitate the reproducibility of the results in other studies.

The mechanisms behind the effect of exercise in the reduction of hyperglycemia are complex and multiple. Both aerobic and strength training are known to improve glycemic control, insulin sensitivity [49–53], insulin responsiveness [54], reduce weight, abdominal fat [51], and BMI [55]. Clearly, all these functions may have played a role in the reduction of fasting and post-prandial hyperglycemia in our study. Declining insulin sensitivity and defects in insulin secretion are crucial markers of the evolution from prediabetes toward diabetes. In fact, in our study, subjects that reverted prediabetes had an improvement in insulin sensitivity. Also, hypertriglyceridemia—a known marker of insulin resistance—ameliorated in patients who reverted (Table 1) [56]. However, there were no differences in weight during follow-up between patients in whom prediabetes reverted or persisted. This may be the consequence of the low number of cases included in the study which may have led to limited power to evaluate weight changes. Be as it may, our study was not designed to evaluate the pathways involved in glucose control due to exercise, an aspect that is worth investigating.

Another relevant aspect of the study was that pro-active surveillance of compliance was crucial to achieve the reversibility of prediabetes. In particular, a baseline plan alone was effective in 5 out 27 (20%) subjects who did not need extra support during the study. In the remaining 22 (80%) at least one extra measure was needed to improve adherence. These results are not explained by treating subjects well predisposed to exercise, since this aspect was not considered in the inclusion criteria. In fact, our results are comparable to those of other studies that implemented active interventions to promote compliance (60–80%) [24–26]. Thus, our study indicates that high levels of compliance are possible to achieve when specific measures are implemented. On the other hand, in most of the patients with bad compliance from the beginning, prediabetes did not revert despite active interventions. Our results highlight the relevance of starting a pro-active intervention on compliance in parallel with the prescription of exercise. Clearly, the results of our study would not have been the same without the design of a simultaneous plan of adherence with the exercise program. Finally, it is important to note that the reversibility of prediabetes may be also achieved with moderate adherence to exercise. In our study, 9 out of 14 (65%) cases with moderate adherence reverted to normal glucose metabolism. These results suggest that in some patients, efficacy can be achieved with a more flexible approach.

The limitations of the study are worth mentioning. The study is exploratory in nature and so, its results must be tested in ad hoc designed clinical trials. Also, the impact of exercise in the prevention of PTDM must be tested specifically. Our population was Caucasian, and so, the impact of exercise in other populations with prediabetes remains to be proven. Finally, the study did not test the effect of diet or specific medication that reduces weight (GLP-1 antagonists) alone or in combination with exercise in the reversibility of prediabetes.

## Conclusion

In conclusion, for renal transplant patients with prediabetes at risk for late PTDM, exercise training was an effective alternative to improve glucose metabolism. Exercise prescription is not a simple procedure and must be conducted focused on individual characteristics. A pre-defined strategy to improve compliance must be considered simultaneously with exercise prescription. Exercise as medicine should be considered as an option for renal transplanted patients at risk for PTDM.

## Data Availability

The authors confirm that the data supporting the findings of this study are available within the article.

## Abbreviations

1 RM: One repetition maximum
BMI: Body mass index
EXPRED: Exercise and prediabetes after renal transplantation
GPAQ: General Physical Activity Questionnaire
HbA1c: Glycated hemoglobin
IFG: Impaired fasting glucose
IGT: Impaired glucose tolerance
OGTT: Oral glucose tolerance test
PTDM: Post-transplant diabetes mellitus
T2DM: Type 2 diabetes mellitus
WHO: World Health Organization
